# Retraction Note: Chinese medicine Di-Huang-Yi-Zhi protects PC12 cells from H_2_O_2_-induced apoptosis by regulating ROS-ASK1-JNK/p38 MAPK signaling

**DOI:** 10.1186/s12906-022-03665-3

**Published:** 2022-07-08

**Authors:** Li-Min Zhang, Rong-Rong Zhen, Chao Gu, Tian-Li Zhang, Yue Li, Miao Jin, Bing Hu, Hong-Mei An

**Affiliations:** 1 Department of Neurology, Longhua Hospital, Shanghai University of Traditional Chinese Medicine, Shanghai, 200032 China; 2 Department of Oncology, Institute of Traditional Chinese Medicine in Oncology, Longhua Hospital, Shanghai University of Traditional Chinese Medicine, Shanghai, 200032 China; 3 Department of Science & Technology, Longhua Hospital, Shanghai University of Traditional Chinese Medicine, Shanghai, 200032 China

**Retraction Note: BMC Complement Med Ther 20, 54 (2020)**

**https://doi.org/10.1186/s12906-020-2834-8**

The Editors have retracted this article. After publication, concerns were raised regarding partial image overlap. Specifically:

- In Fig. 3A, subpanels b and c appear to overlap, with several cells missing from subpanel b.
- In Fig. 5a, subpanels e and f appear to overlap, but have different brightness.

The authors have only been able to retrieve parts of the original data. The Editors therefore no longer have confidence in the data presented in this article.

All authors agree to this retraction.

